# American Society of Anesthesiologists Physical Status Classification as a reliable predictor of postoperative medical complications and mortality following ambulatory surgery: an analysis of 2,089,830 ACS-NSQIP outpatient cases

**DOI:** 10.1186/s12893-021-01256-6

**Published:** 2021-05-21

**Authors:** Colin Foley, Mark C. Kendall, Patricia Apruzzese, Gildasio S. De Oliveira

**Affiliations:** 1 Department of Anesthesiology, The Warren Alpert Medical School of Brown University, 593 Eddy Street, Davol #129, Providence, RI 02903 USA; 2 Department of Anesthesiology, Rhode Island Hospital, Providence, RI USA

**Keywords:** Outpatient surgeries, American society of anesthesiologists physical status (ASA PS), Postoperative outcomes, Ambulatory surgery

## Abstract

**Background:**

Seventy percent of surgical procedures are currently performed in the outpatient setting. Although the American Society of Anesthesiologists (ASA) Physical Classification ability to predict risk has been evaluated for in-patient surgeries, an evaluation in outpatient surgeries has yet to be performed. The major goal of the current study is to determine if the ASA classification is an independent predictor for morbidity and mortality for outpatient surgeries.

**Methods:**

The 2005 through 2016 NSQIP Participant Use Data Files were queried to extract all patients scheduled for outpatient surgery. ASA PS class was the primary independent variable of interest. The primary outcome was 30-day medical complications, defined as having one or more of the following postoperative outcomes: (1) deep vein thrombosis, (2) pulmonary embolism, (3) reintubation, (4) failure to wean from ventilator, (5) renal insufficiency, (6) renal failure, (7) stroke, (8) cardiac arrest, (9) myocardial infarction, (10) pneumonia, (11) urinary tract infection, (12) systemic sepsis or septic shock. Mortality was also evaluated as a separate outcome.

**Results:**

A total of 2,089,830 cases were included in the study. 24,777 (1.19%) patients had medical complications and 1,701 (0.08%) died within 30 days. ASA PS IV patients had a much greater chance of dying when compared to healthy patients, OR (95%CI) of 89 (55 to 143), *P* < 0.001. Nonetheless, over 30,000 ASA PS IV patients had surgery in the outpatient setting. Multivariable analysis demonstrated a stepwise independent association between ASA PS class and medical complications (C statistic = 0.70), mortality (C statistic = 0.74) and readmissions (C statistic = 0.67). Risk stratifying ability was maintained across surgical procedures and anesthesia techniques.

**Conclusions:**

ASA PS class is a simple risk stratification tool for surgeries in the outpatient setting. Patients with higher ASA PS classes subsequently developed medical complications or mortality at a greater frequency than patients with lower ASA PS class after outpatient surgery. Our results suggest that the ambulatory setting may not be able to match the needs of high-risk patients.

**Supplementary Information:**

The online version contains supplementary material available at 10.1186/s12893-021-01256-6.

## Background

The American Society of Anesthesiologists Physical Status (ASA PS) Classification System was first introduced in 1941 [1]. It has been updated over the years and currently consist of 5 categories of PS classification. The ASA classification has been criticized over the years for lack of reliability due to the subjective assessment of its assessments [2]. In addition, the use of intraoperative variables (e.g., surgical duration) has been shown to improve the ability of ASA PS classification to predict adverse postoperative outcomes [3, 4]. Nonetheless, ASA PS class is currently used for any surgical case performed under anesthesia mainly because of its simplicity [5].

Approximately seventy percent of all surgical procedures are currently performed in the ambulatory setting [6, 7]. Several ambulatory surgical centers and state regulations utilize the ASA PS classification to select patients for ambulatory procedures. For example, some ambulatory centers will not perform surgery if the patients’ ASA class is ≥ 3 [8, 9]. Although the ASA PS classification has been evaluated multiple times for inpatient surgeries, the evaluation of ASA PS classification as a predictive tool for morbidity and mortality in outpatient surgeries has yet to be performed.

The major goal of the current study is to evaluate the ASA PS as an independent predictor for morbidity and mortality for ambulatory surgeries. In addition, we sought to explore how the ASA PS classification ability to predict morbidity and/or mortality would vary according to specific surgical procedures and anesthetic techniques. This knowledge would possibly allow more patients to safely undergo ambulatory surgeries.

## Methods

This study was reviewed by the Institutional Review Board committee (#418219) of Rhode Island Hospital (IRB# 1532588) which determined that the study qualified for exemption (45 CFR 46.104(d)) because the deidentified data sets are derived from a publicly available ACS NSQIP database. The NSQIP data user agreement was obtained, and patient informed consent was not applicable due to the exemption status granted by the Institutional Review Board.

All methods were carried out in accordance with relevant guidelines and regulations. Clinical information of the subjects was obtained for the years 2005 through 2016 from the American College of Surgeons (ACS) National Surgical Quality Improvement Program (NSQIP) database. The study is reported following the STROBE guidelines for reporting observational studies [10].

The ACS-NSQIP database is a national prospective database that compiles voluntarily reported data from over 680 institutions in the United States. Over 1 million cases were submitted as part of the 2016 update to the NSQIP database. Data is collected on over 300 variables that include preoperative risk factors, intraoperative variables and post-operative outcomes including complications up to 30 days after surgical procedures. Data collection has been previously described in detail [11, 12]. In brief, data are collected in 8-day cycles, with the first 40 procedures in the cycle included in the dataset. Some commonly performed procedures are capped at 5 within each cycle to increase procedure heterogeneity. Trained clinical nurses assigned at each site collect data for 30 days postoperatively using isolated telephone interviews and operative and clinical notes. Interrater reliability audits of selected participating sites help ensure the collected data are of the highest quality possible. The combined results of inter-rater reliability audits completed to date revealed an overall inter-rater disagreement rate of approximately 1.8% for all assessed program variables [11, 13].

De-identified patient information is freely available to all institutional members who comply with the ACS NSQIP Data Use Agreement. The Data Use Agreement implements the protections afforded by the Health Insurance Portability and Accountability Act of 1996 and the ACS NSQIP Hospital Participation Agreement. The ACS NSQIP and the hospitals participating in this program are the sources of the data used in this study; however, these entities have not verified and are not responsible for the statistical validity of the data analysis or the conclusions derived by the authors.

The 2005 through 2016 NSQIP Participant Use Data Files were queried to extract all patients scheduled. Patients who qualified for the study under these criteria were then separated to an outpatient cohort, defined as length of stay (LOS) of 0 days. Cases described as cardiac and neurosurgery were excluded as those procedures are not routinely done in the outpatient setting. We also excluded trauma, fracture, neoplasms, infectious diseases or patients under 18 years of age.

### Primary Explanatory and Outcome Variables

American Society of Anesthesiologists Physical Status Classification was the primary independent variable of interest and is defined from 1 to 5, where 1 = “a normal healthy patient”, 2 = “a patient with mild systemic disease,” 3 = “a patient with severe systemic disease,” 4 = “a patient with severe systemic disease that is a consistent threat to life.” Patients classified as ASA 5, “a moribund patient who is not expected to survive without the operation” were not included as surgeries for these patients are not usually performed in an outpatient setting.

The primary outcome was 30 day medical complications, defined as having one or more of the following 13 postoperative outcomes: deep vein thrombosis, pulmonary embolism, reintubation, failure to wean from ventilator, renal insufficiency, renal failure, stroke, cardiac arrest, myocardial infarction, pneumonia, urinary tract infection, systemic sepsis or septic shock. Mortality and readmission were also evaluated as separate outcomes of interest.

### Statistical Analysis

The demographic data variables extracted from each data set included age, sex, race, body mass index, ASA PS, smoking status, type of surgery, and clinical characteristics. The surgical categories included general, orthopaedics, urology, gynecological, vascular, plastic and otolaryngology*.* Comorbidities were also recorded and included diabetes mellitus, hypertension, and bleeding disorders. The logarithm of sum relative value units (RVUs) per procedure were used as a measure of surgical complexity. Missing data were analyzed using multiple imputation. Continuous variables were analyzed using independent sample t-tests and categorical variables were analyzed using Chi-Square Test of Independence.

Multiple logistic regression analysis was performed to evaluate ASA PS as an independent predictor of medical complications, hospital readmissions and death. ASA PS class 1 (normal healthy patient) was used as a reference group to determine the relationship between the different ASA PS classes and occurrence of medical complications, hospital readmissions, and death.

ASA PS class was then standardized by CPT code for the overall regression and two subgroup analyses in order to control for interprocedural variability. This was performed by subtracting the mean ASA PS class value for each CPT code from the individual ASA PS class for each case and dividing by the standard deviation of ASA PS for each CPT code. Two subgroup analyses were then performed by multiple logistic regression of individual surgical specialties and the three most common CPT codes. We also examined if the results varied by the type of anesthesia for the procedure: (1) sedation/monitored anesthesia care, (2) regional anesthesia, and (3) general anesthesia.

A two-sided *p* value of ≤ 0.05 was considered to indicate statistical significance. Statistical analyses were performed using SAS software version 9.4 (SAS Institute Inc., Cary, North Carolina).

## Results

**Fig. 1:**
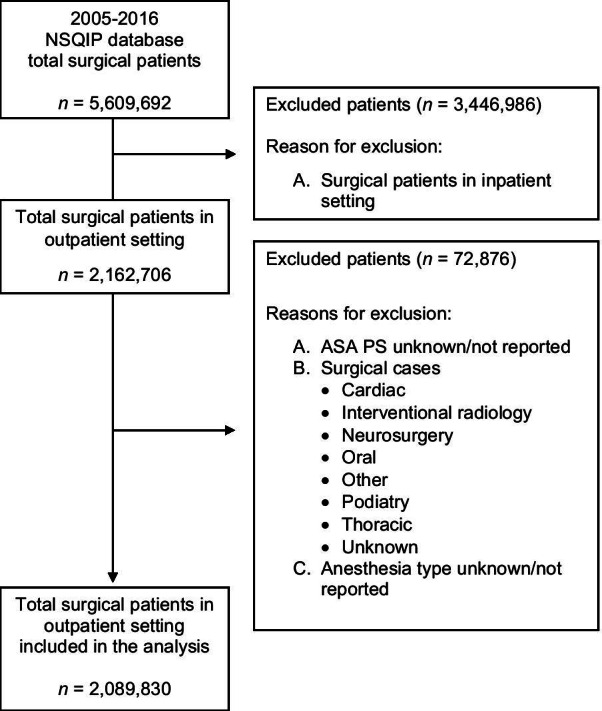
Flow diagram of included and excluded patients. *ASA PS* American Society of Anesthesiologists Physical Status, *NSQIP *National Surgical Quality Improvement. Outpatient setting defined as LOS = 0

The NSQIP database showed 5,609,692 cases in 2005–2016. Of these, 2,162,706 were outpatient cases (Length of stay = 0) and 2,089,830 cases met our inclusion criteria (Fig. 1).

Medical complications and mortality rates by demographic, surgical characteristics (e.g., type and duration) and anesthesia type are presented in Additional file 1: Table S1 and Additional file 2: Table S2, respectively.

**Fig. 2:**
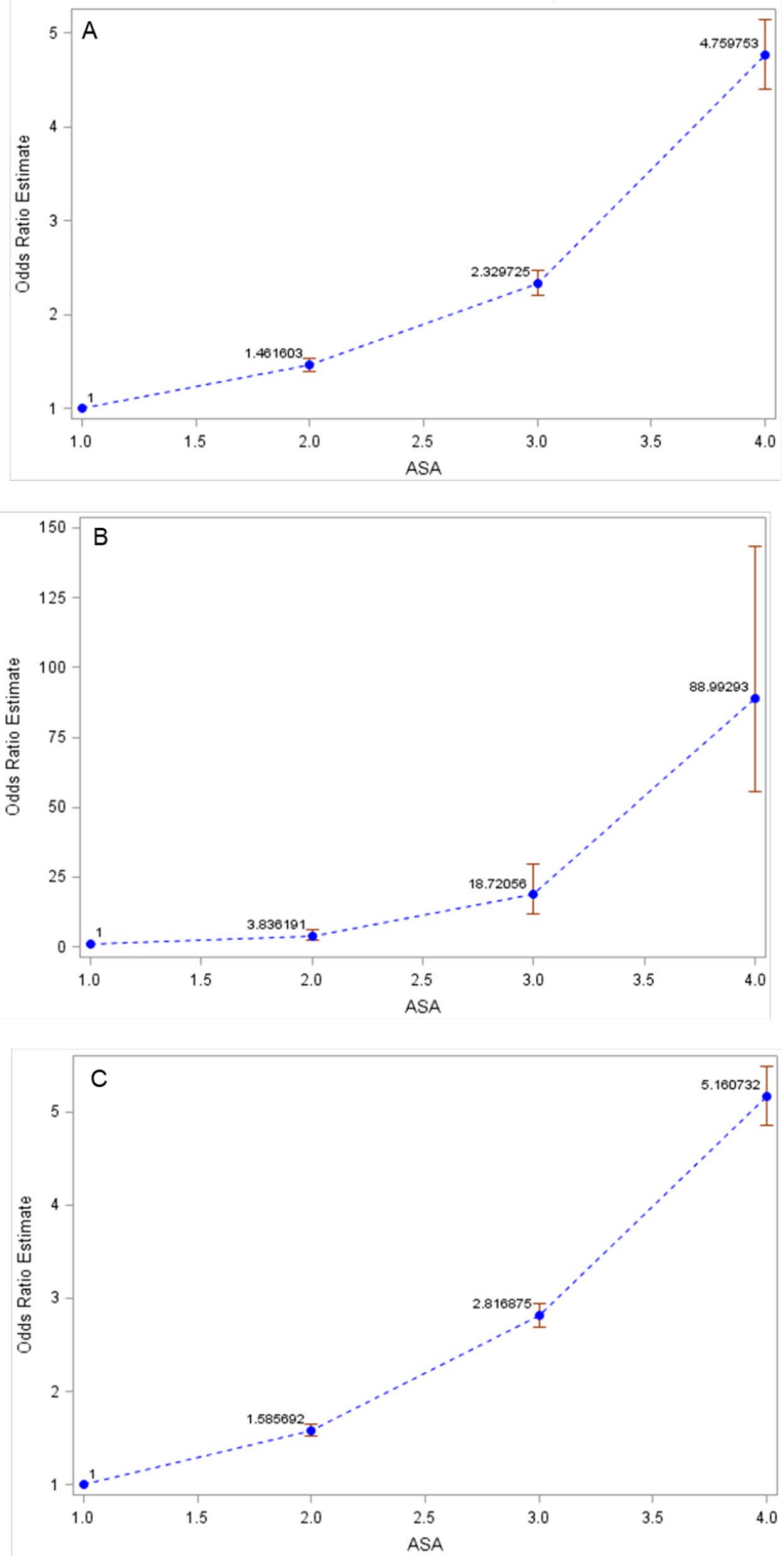
The adjusted odds ratio of medical complications (2A), mortality (2B) and readmissions (2C) increased with greater ASA PS classes in outpatient surgery. Adjusted for gender, smoker, diabetes, dyspnea, obesity, COPD, bleeding disorder, hypertension requiring medication, operative duration, RVU, surgical specialty. Medical complications (C statistic = 0.70), mortality (C statistic = 0.74) and readmission (C statistic = 0.67). Error bars represent 95% confidence interval. ASA PS = American Society of Anesthesiologists Physical Status Classification System

A total of 24,777 (1.19%) patients had medical complications, 40,870 (2.51%) were readmitted to the hospital and 1,701 (0.08%) died within 30 days of the ambulatory surgery. The adjusted odds ratio of medical complications, mortality and readmissions demonstrated a stepwise independent association with ASA PS class (Fig. 2).

An analysis of different specialties using multivariable logistic regression of ASA class Z scores confirmed the association between ASA PS classification and medical complications, readmissions and mortality (Additional file 3: Table S3). In addition, a multivariable analysis of the three most common CPT codes (e.g., laparoscopic cholecystectomy, inguinal hernia and laparoscopic appendectomy) also confirmed the independent association between ASA PS classification and medical complications, readmissions and mortality (Additional file 4: Table S4).

A multivariable regression in patients undergoing outpatient surgery with MAC/Sedation demonstrated a significant role of ASA (Z scores) for medical complications, OR (95% CI) of 1.37 (1.30–1.45) and for mortality, OR (95% CI) of 1.68 (1.48–1.91). In addition, the multivariable regression in patients undergoing outpatient surgery with general anesthesia showed a significant role of ASA (Z scores) for medical complications, OR (95% CI) of 1.25 (1.23–1.27) and mortality, OR (95% CI) of 1.91(1.80–2.03). Similarly, the multivariable analysis or patients undergoing outpatient surgery with regional anesthesia, ASA (Z scores) demonstrated a significant role of ASA (Z scores) for medical complications, OR (95% CI) of 1.26 (1.17, 1.37) and for morality, OR (95%CI) of 1.67 (1.25, 2.24).

## Discussion

The most important finding of this study is that the ASA PS class independently predicts medical complications, readmissions and mortality for surgeries performed in the outpatient setting. Greater ASA PS class was associated with increasing rates of medical complications, readmissions and mortality in many outpatient procedures across surgical subspecialties. There was no overlap in the 95% confidence intervals for medical complications and mortality risk in each ASA PS class further supporting the risk stratifying ability of ASA PS classification. To the best of our knowledge, this is the first study to investigate the ability of ASA PS class to predict medical complications and mortality in patients undergoing outpatient surgery.

Our results are clinically important given the continued trend in healthcare setting from inpatient to outpatient surgery. Given this trend, it is important to validate currently used surgical risk stratification tools in the ambulatory setting. Moreover, reimbursements are increasingly tied to quality metrics and outcomes. Our results ensure that outpatient centers that serve more medically complicated patients have a simple method to adjust for differences in patient complexity when reporting quality outcomes.

Another important finding of our investigation was that the ASA PS class remained a robust predictor of morbidity and mortality across different surgical specialties and anesthesia types. The ASA PS class estimation of mortality and morbidity risk was not significantly changed when anesthesia type and surgical procedures were held constant. In both cases, the ASA PS classification retained no overlap in the 95% confidence intervals for medical complications and mortality risk and remained independently predictive of risk. This supports the generalizability of ASA PS class as a preoperative risk stratifying tool for outpatient surgery.

While the predictive power of ASA PS status for morbidity and mortality appears to remain strong for outpatient surgeries, the magnitude of difference in odds ratios (risk) between each increasing ASA PS level was slightly smaller than in a prior study that examined inpatient surgeries [14]. This suggests that other factors beyond medical covariates may influence medical complication and mortality after ambulatory surgery. For example, since patients must provide self-care after ambulatory surgery, it is possible that poor health literacy may be associated with adverse outcomes [15, 16]. In addition, family support may also be a factor determining outcomes, especially for more vulnerable patients (e.g., seniors) [17].

It was also interesting to note that over 34,000 patients classified as ASA PS 4 had ambulatory surgery. After adjusting for potential confounding factors, ASA PS 4 patients had an 89 times greater chance of mortality after ambulatory surgery when compared to ASA PS 1 (healthy) patients. Our results suggest that the ambulatory setting may not be able to match the needs of high-risk patients. These patients are probably better suited for surgery in the inpatient setting where close monitoring, potent IV medications and nursing care are readily available during the postoperative period [18, 19].

The preoperative evaluation and risk stratification, including the prediction and prevention of adverse outcomes in the perioperative period is paramount to the safe and effective practice of perioperative medicine [20]. Efforts have also been made to create risk calculators with multiple variables, however these have proved complicated and therefore have not been widely implemented [21, 22]. In addition, lack of pertinent data across all surgical settings and anesthesia techniques have limited their generalizability. Given these deficiencies, our study demonstrates that the ASA PS class is a simple and acceptable method to accurately risk stratify patients undergoing outpatient surgery across multiple specialties and anesthesia techniques.

Our study can only be interpreted within the context of its limitations. Similar to prior studies, we used RVUs to account for complexity of surgical cases and we used all types of procedures performed in each specialty [23, 24]. Nevertheless, it is possible that certain procedures within a subspecialty could themselves be independent risk factors for medical complications and/or mortality. We attempted to address this by performing subspecialty group analysis and a subgroup analysis of most common CPT codes. Lastly, we excluded procedures that are not commonly performed on an outpatient basis (e.g., cardiac surgery and neurosurgery), thus results cannot be generalized across these patient groups.

## Conclusion

This study supports the use of the ASA PS class as a simple risk stratification tool for surgeries performed in the ambulatory setting. Patients with higher ASA PS class subsequently developed medical complications or mortality at a greater frequency than patients with lower ASA PS class after outpatient surgery. ASA PS class remained predictive in different specialties and with varying anesthetic techniques. ASA PS IV patients had 89 times greater chance of dying after ambulatory surgery when compared to healthy patients. Nonetheless, over 30,000 ASA PS IV patients had surgery in the outpatient setting.

## Supplementary Information

**Additional file 1. Table S1.** Demographic, surgical characteristics and anesthesia type by medical complications status in patients who underwent outpatient surgery.

**Additional file 2. Table S2.** Demographic, surgical characteristics and anesthesia type by mortality in patients who underwent outpatient surgery.

**Additional file 3. Table S3.** Multivariable logistic regression analysis for medical complications, mortality and readmissions for Z scores of ASA physical status shown by surgical specialty in patients who underwent outpatient surgery.

**Additional file 4. Table S4.** Multivariable logistic regression analysis for medical complications, mortality and readmissions of ASA physical status Z scores for the 3 most common current procedural terminology codes in patients who underwent outpatient surgery.

## Data Availability

The American College of Surgeons National Surgical Quality Improvement Program and the hospitals participating in the ACS NSQIP are the source of the data used herein; they have not verified and are not responsible for the statistical validity of the data analysis or the conclusions derived by the authors. The data that support the findings of this study are available from ACS NSQIP but restrictions apply to the availability of these data, which were used under license for the current study, and so are not publicly available. Data are however available from the authors upon reasonable request and with permission of ACS NSQIP. The URL is https://www.facs.org/Quality-Programs/ACS-NSQIP/participant-use.

## Abbreviations

ACS: American College of Surgeons
ASA PS: American Society of Anesthesiologists Physical Status Classification
CI: Confidence interval
CPT: Current procedural terminology
IRB: Institutional review board
LOS: Length of stay
NSQIP: National surgical quality improvement program database
OR: Odds ratio
RVU: Relative value unit
STROBE: Strengthening the reporting of observational studies in epidemiology
