# Supplementary material for: American Society of Anesthesiologists Physical Status Classification as a reliable predictor of postoperative medical complications and mortality following ambulatory surgery: an analysis of 2,089,830 ACS-NSQIP outpatient cases
Source: BMC Surg. 2021 May 21;21:253. doi: 10.1186/s12893-021-01256-6 (PMC8140433; doi:10.1186/s12893-021-01256-6)
Supplement: Supplementary file 1 — Additional file 1. Table S1. Demographic, surgical characteristics and anesthesia type by medical complications status in patients who underwent outpatient surgery. [file 12893_2021_1256_MOESM1_ESM.pdf]

Table 1. Demographic, Surgical Characteristics and Anesthesia Type by Medical Complications Status in Patients who Underwent Outpatient Surgery

	Cases with No Complications (n=2,065,053)	Cases with Complications (n=24,777)	All Cases	P Value	%TC
**Age,** (y), mean ±SD	51.7 ± 16.5	58.0 ±17.2	51.8 ± 16.5	<.001	
**Operative Time**, (m), mean ±SD	69.6 ± 53.8	84.6 ± 71.4	69.8 ± 54.1	<.001	
**RVU,** mean ±SD	13.6 ±8.7	16.0 ± 11.7	13.6 ± 8.8	<.001	
**Gender,** % (n)					
Female	58.6 (1210929/2063873)	58.9 (14591/24764)	58.7 (1225520/2088637)	0.432	1.19
Male	41.3 (852944/2063873)	41.1 (10173/24764)	41.3 (863117/2088637)	0.432	1.18
**Race,** % (n)		.			
White	84.5 (1530200/1809948)	85.7 (19102/22302)	84.6 (1549302/1832250)	<.001	1.23
Black	10.6 (191964/1809948)	10.0 (2229/22302)	10.6 (194193/1832250)	0.003	1.15
Asian	3.2 (57547/1809948)	2.6 (579/22302)	3.2 (58126/1832250)	<.001	1.00
Other	1.7 (30237/1809948)	1.8 (392/22302)	1.7 (30629/1832250)	0.313	1.28
**Smoker,** % (n)	17.5 (361511/2065042)	18.3 (4528/24777)	17.5 (366039/2089819)	0.002	1.24
**Diabetes,** % (n)	10.7 (220179/2065050)	18.3 (4536/24777)	10.8 (224715/2089827)	<.001	2.02
**Dyspnea,** % (n)	4.3 (87677/2065045)	8.5 (2105/24777)	4.3 (89782/2089822)	<.001	2.34
**Obesity,** % (n)	40.3 (819842/2034300)	44.4 (10830/24408)	40.4 (830672/2058708)	<.001	1.30
**COPD,** % (n)	2.5 (51857/2065049)	6.3 (1567/24777)	2.6 (53424/2089826)	<.001	2.93
**Bleeding disorder,** % (n)	1.8 (37504/2065050)	4.9 (1205/24777)	1.9 (38709/2089827)	<.001	3.11
**Hypertension,** % (n)	35.1 (725061/2065050)	49.0 (12136/24777)	35.3 (737197/2089827)	<.001	1.65
ASA PS 1	16.0 (330417/2065053)	7.3 (1807/24777)	15.9 (332224/2089830)	<.001	0.54
ASA PS 2	56.5 (1166831/2065053)	45.3 (11224/24777)	56.4 (1178055/2089830)	<.001	0.95

ASA PS 3	25.9 (534666/2065053)	41.1 (10173/24777)	26.1 (544839/2089830)	<.001	1.87
ASA PS 4	1.6 (33139/2065053)	6.4 (1573/24777)	1.7 (34712/2089830)	<.001	4.53
**Surgical Specialty,** % (n)					
General surgery	60.1 (1240965/2065053)	47.1 (11661/24777)	59.9 (1252626/2089830)	<.001	0.93
Gynecology	7.8 (161642/2065053)	17.3 (4295/24777)	7.9 (165937/2089830)	<.001	2.59
Orthopedics	14.3 (296146/065053)	8.2 (2029/24777)	14.3 (298175/2089830)	<.001	0.68
ENT	4.4 (91314/2065053)	2.4 (594/24777)	4.4 (91908/2089830)	<.001	0.65
Plastic surgery	4.6 (94009/2065053)	2.2 (543/24777)	4.5 (94552/2089830)	<.001	0.57
Urology	5.2 (107612/2065053)	16.6 (4120/24777)	5.4 (111732/2089830)	<.001	3.69
Vascular	3.6 (73365/2065053)	6.2 (1535/24777)	3.6 (74900/2089830)	<.001	2.05
**Anesthesia Type,** % (n)					
General Anesthesia	89.1 (1840197/2065053)	90.7 (22466/24777)	89.1 (1862663/2089830)	<.001	1.21
Neuroaxial/Regional	2.1 (43029/2065053)	3.1 (778/24777)	2.10 (43807/2089830)	<.001	1.78
MAC/IV Sedation/Local	8.8 (181827/2065053)	6.2 (1533/24777)	8.8 (183360/2089830)	<.001	0.84

* $\chi 2$ test for binary variables, independent sample t-test for continuous variables.

ASA PS = American Society of Anesthesiologists physical status classification system, COPD = chronic obstructive pulmonary disease, IV = intravenous, MAC = monitored anesthesia care, TC = percent of total with complications, RVU = relative value units

Outpatient surgery defined as length of stay = 0 days.
