# Supplementary material for: American Society of Anesthesiologists Physical Status Classification as a reliable predictor of postoperative medical complications and mortality following ambulatory surgery: an analysis of 2,089,830 ACS-NSQIP outpatient cases
Source: BMC Surg. 2021 May 21;21:253. doi: 10.1186/s12893-021-01256-6 (PMC8140433; doi:10.1186/s12893-021-01256-6)
Supplement: Supplementary file 2 — Additional file 2. Table S2. Demographic, surgical characteristics and anesthesia type by mortality in patients who underwent outpatient surgery. [file 12893_2021_1256_MOESM2_ESM.pdf]

Table 2. Demographic, Surgical Characteristics and Anesthesia Type by Mortality in Patients who Underwent Outpatient Surgery

| | Cases with No Death (n=2088129) | Cases with Death (n=1701) | All Cases | P Value | %TC |
|---|---|---|---|---|---|
| **Age, (y),** mean ±SD | 51.8 ± 16.48 | 69.0 ± 14.68 | 51.8 ± 16.49 | <.001 | |
| **Operative Time, (m),** mean ±SD | 69.8 ± 54.05 | 83.9 ± 78.08 | 69.8 ± 54.07 | <.001 | |
| **RVU,** mean ±SD | 13.6 ± 8.75 | 16.7 ± 14.28 | 13.6 ± 8.76 | <.001 | |
| Female | 58.7 (1224789/2086936) | 43.0 (731/1701) | 58.7 (1225520/2088637) | <.001 | 0.06 |
| Male | 41.3 (862147/2086936) | 57.0 (970/1701) | 41.3 (863117/2088637) | <.001 | 0.11 |
| **Race,** % (n) | | | | | |
| White | 84.6 (1547945/1830672) | 86.0 (1357/1578) | 84.6 (1549302/1832250) | 0.114 | 0.09 |
| Black | 10.6 (194022/1830672) | 10.8 (171/1578) | 10.6 (194193/1832250) | 0.759 | 0.09 |
| Asian | 3.2 (58090/1830672) | 2.3 (36/1578) | 3.2 (58126/1832250) | 0.043 | 0.06 |
| Other | 1.7 (30615/1830672) | 0.9 (14/1578) | 1.7 (30629/1832250) | 0.015 | 0.05 |
| **Smoker,** % (n) | 17.5 (365709/2088118) | 19.4 (330/1701) | 17.5 (366039/2089819) | 0.041 | 0.09 |
| **Diabetes**, % (n) | 10.7 (224235/2088126) | 28.2 (480/1701) | 10.8 (224715/2089827) | <.001 | 0.21 |
| **Dyspnea,** % (n) | 4.3 (89438/2088121) | 20.2 (344/1701) | 4.3 (89782/2089822) | <.001 | 0.38 |
| **Obesity,** % (n) | 40.4 (830109/2057075) | 34.5 (563/1633) | 40.4 (830672/2058708) | <.001 | 0.07 |
| **COPD,** % (n) | 2.6 (53170/2088125) | 14.9 (254/1701) | 2.6 (53424/2089826) | <.001 | 0.48 |
| **Bleeding disorder,** % (n) | 1.8 (38479/2088126) | 13.5 (230/1701) | 1.9 (38709/2089827) | <.001 | 0.59 |
| **Hypertension,** % (n) | 35.3 (736022/2088126) | 69.1 (1175/1701) | 35.3 (737197/2089827) | <.001 | 0.16 |
| ASA PS 1 | 15.9 (332204/2088129) | 1.2 (20/1701) | 15.9 (332224/2089830) | <.001 | 0.01 |
| ASA PS 2 | 56.4 (1177762/2088129) | 17.2 (293/1701) | 56.4 (1178055/2089830) | <.001 | 0.02 |
| ASA PS 3 | 26.1 (543933/2088129) | 53.3 (906/1701) | 26.1 (544839/2089830) | <.001 | 0.17 |
| ASA PS 4 | 1.6 (34230/2088129) | 28.3 (482/1701) | 1.7 (34712/2089830) | <.001 | 1.39 |
| **Surgical Specialty,** % (n) | | | | | |

| | | | | | |
|---|---|---|---|---|---|
| General surgery | 59.9 (1251680/2088129) | 55.6 (946/1701) | 59.9 (1252626/2089830) | <.001 | 0.08 |
| Gynecology | 7.9 (165897/2088129) | 2.4 (40/1701) | 7.9 (165937/2089830) | <.001 | 0.02 |
| Orthopedics | 14.3 (298057/2088129) | 6.9 (118/1701) | 14.3 (298175/2089830) | <.001 | 0.04 |
| ENT | 4.4 (91856/2088129) | 3.1 (52/1701) | 4.4 (91908/2089830) | 0.007 | 0.06 |
| Plastic surgery | 4.5 (94525/2088129) | 1.6 (27/1701) | 4.5 (94552/2089830) | <.001 | 0.03 |
| Urology | 5.3 (111517/2088129) | 12.6 (215/1701) | 5.4 (111732/2089830) | <.001 | 0.19 |
| Vascular | 3.6 (74597/2088129) | 17.8 (303/1701) | 3.6 (74900/2089830) | <.001 | 0.40 |
| **Anesthesia Type,** % (n) | | | | | |
| General Anesthesia | 89.1 (1861289/2088129) | 80.8 (1374/1701) | 89.1 (1862663/2089830) | <.001 | 0.07 |
| Neuroaxial/Regional | 2.1 (43749/2088129) | 3.4 (58/1701) | 2.1 (43807/2089830) | <.001 | 0.13 |
| MAC/IV Sedation/Local | 8.8 (183091/2088129) | 15.8 (269/1701) | 8.8 (183360/2089830) | <.001 | 0.15 |

* $\chi 2$ test was used for binary variables, independent sample t-test was used for continuous variables.
ASA PS = American Society of Anesthesiologists physical status classification system, COPD = chronic obstructive pulmonary disease, ENT = ear, nose and throat, IV = intravenous, MAC = monitored *anesthesia* care, TC = percent of total with complications, RVU = relative value units. Outpatient surgery defined as length of stay = 0 days.
