# Supplementary material for: American Society of Anesthesiologists Physical Status Classification as a reliable predictor of postoperative medical complications and mortality following ambulatory surgery: an analysis of 2,089,830 ACS-NSQIP outpatient cases
Source: BMC Surg. 2021 May 21;21:253. doi: 10.1186/s12893-021-01256-6 (PMC8140433; doi:10.1186/s12893-021-01256-6)
Supplement: Supplementary file 3 — Additional file 3. Table S3. Multivariable logistic regression analysis for medical complications, mortality and readmissions for Z scores of ASA physical status shown by surgical specialty in patients who underwent outpatient surgery. [file 12893_2021_1256_MOESM3_ESM.pdf]

Table 3. Multivariable Logistic Regression Analysis for Medical Complications, Mortality and Readmissions for Z Scores of ASA physical status shown by Surgical Specialty in Patients who Underwent Outpatient Surgery

	# Events	Total n, (%)	Odds Ratio (95% CI)*	P Value*
**Medical Complications**				
General Surgery	11661	1252626 (0.93)	1.38 (1.36, 1.42)	<.0001
Gynecology	4295	165937 (2.59)	1.14 (1.09, 1.17)	<.0001
Orthopedics	2029	298175 (0.68)	1.21 (1.15, 1.27)	<.0001
ENT	594	91908 (0.65)	1.38 (1.26, 1.51)	<.0001
Plastic Surgery	543	94552 (0.57)	1.17 (1.06, 1.28)	0.0012
Urology	4120	111732 (3.69)	1.07 (1.04, 1.11)	<.0001
Vascular	1535	74900, (2.05)	1.26 (1.19, 1.32)	<.0001
**Death**				
General Surgery	946	1252626 (0.08)	2.04 (1.89, 2.19)	<.0001
Gynecology	40	165937 (0.02)	1.67 (1.19, 2.35)	0.0033
Orthopedics	118	298175 (0.04)	1.77 (1.43, 2.18)	<.0001
ENT	52	91908 (0.06)	2.03 (1.50, 2.75)	<.0001
Plastic Surgery	27	94552 (0.03)	1.76 (1.17, 2.66)	0.0070
Urology	215	111732 (0.19)	1.98 (1.70, 2.30)	<.0001
Vascular	303	74900 (0.40)	1.44 (1.27, 1.64)	<.0001
**Readmission**				
General Surgery	23334	893472 (2.61)	1.29 (1.27, 1.31)	<.0001
Gynecology	3359	153669 (2.19)	1.19 (1.15, 1.23)	<.0001
Orthopedics	3327	266932 (1.25)	1.29 (1.24, 1.35)	<.0001
ENT	1834	80602 (2.28)	1.22 (1.16, 1.28)	<.0001
Plastic Surgery	1809	83717 (2.16)	1.25 (1.19, 1.31)	<.0001

Urology	4333	100097 (4.33)	1.23 (1.19, 1.27)	<.0001
Vascular	2874	52750 (5.45)	1.14 (1.10, 1.19)	<.0001

* Results based on multivariable logistic regression adjusted for gender, smoker, diabetes, dyspnea, obesity, COPD, bleeding disorder, hypertension required medication, operative duration, and RVU.
ASA = American Society of Anesthesiologists, ENT = ear, nose and throat. Outpatient surgery defined as length of stay = 0 days.
