# Supplementary material for: American Society of Anesthesiologists Physical Status Classification as a reliable predictor of postoperative medical complications and mortality following ambulatory surgery: an analysis of 2,089,830 ACS-NSQIP outpatient cases
Source: BMC Surg. 2021 May 21;21:253. doi: 10.1186/s12893-021-01256-6 (PMC8140433; doi:10.1186/s12893-021-01256-6)
Supplement: Supplementary file 4 — Additional file 4. Table S4. Multivariable logistic regression analysis for medical complications, mortality and readmissions of ASA physical status Z scores for the 3 most common current procedural terminology codes in patients who underwent outpatient surgery. [file 12893_2021_1256_MOESM4_ESM.pdf]

Table 4. Multivariable Logistic Regression Analysis for Medical Complications, Mortality and Readmissions of ASA physical status Z Scores for the 3 Most Common Current Procedural Terminology Codes in Patients who Underwent Outpatient Surgery

| | CPT code | # Events | Total n, % | Odds Ratio (95% CI)* | P Value* |
|---|---|---|---|---|---|
| **Medical Complications** | | | | | |
| Laparoscopic Cholecystectomy | 47562 | 1662 | 164168, (1.01) | 1.46 (1.38, 1.54) | <.0001 |
| Inguinal Hernia | 49505 | 744 | 126974, (0.59) | 1.65 (1.52, 1.79) | <.0001 |
| Laparoscopic Appendectomy | 44970 | 1343 | 98715, (1.36) | 1.18 (1.11, 1.26) | <.0001 |
| **Death** | | | | | |
| Laparoscopic Cholecystectomy | 47562 | 97 | 164168, (0.06) | 2.24 (1.79, 2.81) | <.0001 |
| Inguinal Hernia | 49505 | 89 | 126974, (0.07) | 2.56 (2.02, 3.24) | <.0001 |
| Laparoscopic Appendectomy | 44970 | 8 | 98715, (0.01) | 3.63 (1.84, 7.13) | 0.0002 |
| **Readmission** | | | | | |
| Laparoscopic Cholecystectomy | 47562 | 3403 | 118451, (2.87) | 1.2678 (1.21, 1.32) | <.0001 |
| Inguinal Hernia | 49505 | 1205 | 84634, (1.42) | 1.84 (1.72, 1.97) | <.0001 |
| Laparoscopic Appendectomy | 44970 | 1814 | 81324, (2.23) | 1.20 (1.14, 1.27) | <.0001 |

*Results based on multivariable logistic regression adjusted for gender, smoker, diabetes, dyspnea, obesity, COPD, bleeding disorder, hypertension required medication, operative duration, and RVU.
ASA = American Society of Anesthesiologists, CPT = Current Procedural Terminology, CI = Confidence interval
Outpatient surgery defined as length of stay = 0 days.
